# Differences in drug use between men and women: an Italian cross sectional study

**DOI:** 10.1186/s12905-017-0424-9

**Published:** 2017-09-05

**Authors:** Daria Putignano, Dario Bruzzese, Valentina Orlando, Denise Fiorentino, Alessia Tettamanti, Enrica Menditto

**Affiliations:** 1 CIRFF, Center of Pharmacoeconomics, Federico II University of Naples, via Domenico Montesano 49, Naples, Italy; 2 Department of Public Health, Federico II University of Naples, Via Pansini, 5, Naples, 80131 Italy; 3 QuintilesIMS RWE , Milan, Italy

**Keywords:** Drug use, Man, Woman, Prevalence, Prescription

## Abstract

**Background:**

Drugs are the most important treatment option for most diseases, and the majority of medical consultations result in a prescription. Women and men receive different drug prescriptions and differ in therapeutic response to pharmacological therapy. This disparity is due to biological factors (sex differences) or/and behavior, lifestyle and life experience (gender differences). Sex differences in drug use have been demonstrated in several therapeutic areas; however, there is a lack of overviews on sex and gender differences of drug use in an entire population.

**Methods:**

We conducted a descriptive cross - sectional drug use study, involving the entire Italian population in 2012, aimed at showing and analyzing differences between men and women as regards their exposure to drugs. The data source was IMS LifeLink Treatment DynamicsTMLRx Database and it included all prescribed drugs reimbursed by the Italian National Healthcare System in 2012 and covered 90% of the entire Italian population.

The information about the prescriptions was stratified by men and women and age. Drug consumption was expressed as DDD/ 1000 ab die. Exposure to drug prescriptions was expressed as period prevalence (the proportion of the population dispensed ≥1 prescription in 2012 per 1000 inhabitants). Differences of prevalence between men and women were expressed as crude and age adjusted risk ratios with 95% CI.

**Results:**

Our findings suggested that the largest differences in drug prescriptions regarded drugs affecting bone structure and mineralization (RR 15.9), calcium (RR 8.6) and thyroid therapy (RR 5.4), dispensed more to women than men. Otherwise ACE inhibitors were more commonly used in men.

**Conclusions:**

This is the first study exploring difference in drug use between men and women and carried out on the entire Italian population. Our findings showed substantial differences between men and women in term of prevalence of drug prescriptions. Some differences in drug use may be explained by sex differences (variations in disease prevalence and severity, pathophysiology, or by other biological differences), other differences need further investigation to explain the apparent lack of a rational medical explanation for some findings.

The findings may subsequently be used to plan future studies to address differences suggesting inequity in treatment approaches.

**Electronic supplementary material:**

The online version of this article doi:(10.1186/s12905-017-0424-9) contains supplementary material, which is available to authorized users.

## Background

Drugs are the most important treatment option for most diseases and, the majority of medical consultations result in a prescription [1]. Women and men receive different drug prescriptions and differ in therapeutic response to pharmacological therapy [2–5]. This disparity between men and women is most apparent in the population ranging from 45 to 64 years of age; an age when women’s health issues primarily revolve around chronic conditions [5].

In order to discuss the differences between men and women as regards drug prescription, it is necessary to distinguish between sex and gender. Sex differences are based on biological factors (reproductive function, concentrations of sex hormones, expression of genes on X and Y chromosomes and their effects and the higher percentage of body fat in women). By contrast, gender is associated with behavior, lifestyle and life experience. Both sex and gender may be considered as a factor in the prescription of drugs, yet, while it is evident that sex differences should be considered when prescribing medicines, it is unclear as to what gender differences should be considered by the prescribing physician.

It is known that women are more likely to use several classes of medications including antidepressants and anti-anxiety and pain medications, while men use more cardiovascular medications than do women [6]. In addition, sex differences in drug use have been demonstrated in several therapeutic areas [7–11]. However, there is a lack of overviews regarding both sex and gender differences concerning the use of drugs in an entire population. We have assumed, therefore, that a study of drug use based on information obtained from administrative databases [12–14] represents a suitable tool for the evaluation of use of drugs by women and men.

## Methods

### Aim, design and setting

We conducted a descriptive cross - sectional drug use study aimed at showing and analyzing differences between men and women in the entire Italian population in 2012 as regards exposure to drugs.

### Data source

LifeLink Treatment DynamicsTMLRx Database IMS Health was the data source for this study. It is a database containing all the information (>90% coverage) concerning the drugs dispensed in Italy in 2012 and fully reimbursed by the National Health Service (NHS). All the drugs provided by private and public pharmacies to residents in Italy on prescription and within the limits of the benefits payable by the NHS were recognized. The information concerning the prescriptions (drug, date of dispensing) were stratified by gender and age of patients. Each patient was identified by an anonymous code which made it impossible for researchers to identify any patient. Demographic data used as denominators of indicators of exposure to pharmaceutical prescription were retrieved from the ISTAT website.

The above database has been extensively used for drug use studies [15, 16].

### Case definition

In 2012, all patients who received at least one prescription in less than 12 months were defined users or *prevalent cases* and included in this study.

### Drugs

Drugs included in the analysis were identified by means of the Anatomical Therapeutic Chemical (ATC) classification. The ATC system classifies drugs into five hierarchical levels based on the organs or systems on which they act and on their chemical, pharmacological and therapeutic properties. At the first level (ATC I), drugs are divided into fourteen main anatomical groups and, within these, into the main therapeutic groups (ATC II). The third (ATC III) and fourth (ATC IV) levels are chemical/ pharmacological/therapeutic subgroups. In the fifth and final level, the individual substances are classified.

**Fig. 1:**
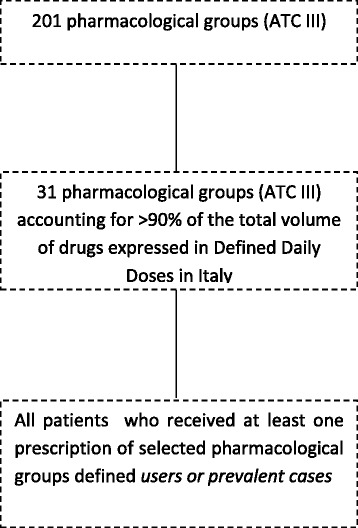
Flow – chart

In this study, all the pharmacological groups (ATC III), accounting for >90% of the total volume expressed in Defined Daily Doses were selected. Therefore, we analysed 31 pharmacological groups (Fig. 1).

### Outcomes and statistical analysis

Drug consumption was expressed as DDD/ 1000 ab die. DDD is the assumed *average maintenance dose per day for a drug used for its main indication in adults.* The number of DDDs is reported as per 1000 inhabitants per day (DDD/1000 inhabitants/day or DID).

Exposure to drug prescriptions was defined as the period prevalence (proportion of the population in the country dispensed ≥ 1 prescription in 2012 per 1000 inhabitants).

Differences of prevalence between men and women were expressed as crude and age adjusted risk ratios (RR) with 95% Confidence Interval (CI) (ratio of the prevalence in women and men). Age standardization was performed by direct standardization where the Italian population on 31 December 2011 (29,413,274 men and 31,213,168 women) was used as the standard population. Ninety-five percent confidence interval of crude and age adjusted risk ratios were computed using standard methods [17]. Data were analyzed using the R software version 3.2.0.

## Results

The volume of drugs belonging to the 31 pharmacological group included in this study and dispensed in Italy in 2012 was 19 billion DDD, corresponding to 888.4 DDD/1000 inhabitants daily. The analysis of the prevalence of drug prescription stratified by age group showed that antibiotics such as penicillins (ATC: J01C 28.1‰), drugs for obstructive airway diseases (ATC: R03B 13.5‰) and antihistamines for systemic use (ATC: R06A 12.3‰) were the most common drugs used by children and adolescents for both men and women. Antibiotics remained the most used pharmacological group in prevalent cases until 59 years old (25.6‰) followed by non-steroidal anti-inflammatory drugs (ATC: M01A 16.0‰). Prescription of gastrointestinal and cardiovascular drugs increased starting from 60 years old.

In all age groups, women had a higher prescription prevalence for most pharmacological groups except for drugs used in benign prostatic hypertrophy, anti-gout preparations and drugs used to treat cardiovascular disease (including anti-lipemic agents, beta- blockers and related medicines and angiotensin – converting enzyme inhibitors). Drugs affecting bone structure and mineralization (31.7‰ women vs 2.0‰ men), calcium (24.3‰ women vs 2.8‰ men) and thyroid preparations (64.8‰ women vs 12.0‰ men) had a higher prevalence of use in women than men as did iron preparations (10.1‰ women vs 3.8‰ men), vitamin B12 and folic acid (7.0‰ women vs 3.7‰ men) and antidepressants (68.0‰ women vs 29.5‰ men). On the contrary, drugs used in benign prostatic hypertrophy (61.9‰ men), anti-gout (11.5‰ women vs 20.9‰ men) and Angiotensin-converting enzyme inhibitors (ACE inhibitors) (58.6‰ women vs 70.2‰ men) were more used for men than for women (Table 1) Additional file 1.

**Table 1:** Prevalence of use (‰) of the 31 most common pharmacological groups in 2012 in Italy

ATC III	Description	Prevalence (‰)		Crude Relative Risk	Adjusted Relative Risk
		Male	Female		
A02B	Drugs for peptic ulcer and gastro-oesophageal reflux disease (GORD)	164,1	128,2	1,3	1,1
A10A	Insulins and analogues	10,5	10,9	1,0	0,8
A10B	Blood glucose lowering drugs, excluding insulins	45,7	50,6	0,9	0,8
A12A	Calcium	24,3	2,8	8,6	7,0
B01A	Antithrombotic agents	106,5	113,9	0,9	0,7
B03A	Iron preparations	10,1	3,8	2,6	2,1
B03B	Vitamin B12 and folic acid	7,0	3,7	1,9	1,6
C01B	Antiarrhythmics, class I and III	10,1	10,6	1,0	0,7
C01D	Vasodilators used in cardiac diseases	14,5	15,0	1,0	0,7
C02C	Antiadrenergic agents, peripherally acting	11,9	13,4	0,9	0,7
C03C	High-ceiling diuretics	34,0	26,4	1,3	0,9
C07A	Beta blocking agents	84,8	72,1	1,2	1,0
C08C	Selective calcium channel blockers with mainly vascular effects	52,6	57,7	0,9	0,7
C09A	ACE inhibitors, plain	58,6	70,2	0,8	0,7
C09B	ACE inhibitors, combinations	44,7	36,1	1,2	1,0
C09C	Angiotensin II antagonists, plain	49,0	46,8	1,1	0,9
C09D	Angiotensin II antagonists, combinations	62,1	46,3	1,4	1,1
C10A	Lipid modifying agents, plain	91,4	97,8	0,9	0,8
G04C	Drugs used in benign prostatic hypertrophy	0,2	61,9	0,0	0,0
H02A	Corticosteroids for systemic use, plain	32,9	23,2	1,4	1,3
H03A	Thyroid preparations	64,8	12,0	5,4	5,0
J01C	Beta-lactam antibacterials, penicillins	71,2	53,4	1,3	1,3
M01A	Anti-inflammatory and antirheumatic products, non-steroids	124,5	73,9	1,7	1,5
M04A	Antigout preparations	11,5	20,9	0,6	0,4
M05B	Drugs affecting bone structure and mineralization	31,7	2,0	16,0	12,5
N03A	Antiepileptics	21,6	18,7	1,2	1,1
N06A	Antidepressant	68,0	29,5	2,3	2,0
R03A	Adrenergics, inhalants	30,3	33,3	0,9	0,8
R03B	Other drugs for obstructive airway diseases, inhalants	27,6	28,6	1,0	0,8
R06A	Antihistamines for systemic use	26,4	20,8	1,3	1,3
S01E	Antiglaucoma preparations and miotics	21,2	17,0	1,3	1,0

**Fig. 2:**
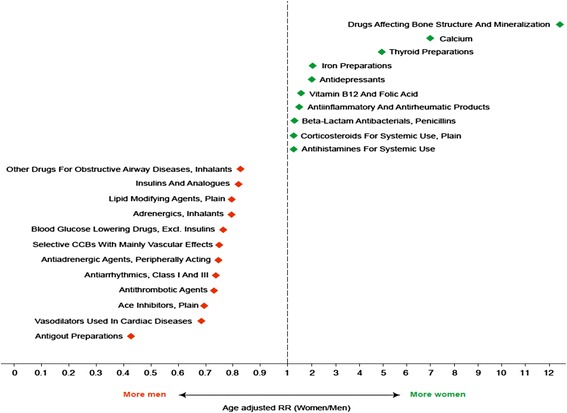
Pharmacological groups with the highest age- adjusted relative differences in prevalence in 2012 in Italy

The crude differences between men and women for the 31 therapeutic categories in the study, expressed in terms of prevalence, were statistically significant. Overall, after age adjustment, differences remained. The large differences in drugs used for bone disease such as osteoporosis diminished after age adjustment, even though they were still more common in women. The pharmacological groups with the largest relative differences, and more commonly dispensed to women, were drugs affecting bone structure and mineralization (RR 15.9), calcium (RR 8.6), thyroid preparations (RR 5.4), iron preparations (RR 2.6), antidepressants (RR 2.3) and anti-anemic preparations (RR 2.0) (Fig. 2).

## Discussion

To the best of our knowledge, this is the first study exploring gender difference in drug use and carried out on the entire Italian population. Our findings showed substantial differences between men and women in terms of prevalence of drug prescriptions. The volume of drugs belonging to the pharmacological groups in the study which were dispensed in Italy in 2012 was 19 billion DDD corresponding to 888.4 DDD/1000 inhabitants daily. Antibiotics and anti-asthmatics were the most used drugs in Italian children and adolescents for both males and females. These results were consistent with another national drug use study in which antibiotics were widely prescribed in pediatric outpatients with both quantitative and qualitative marked territorial differences [18].

Our results highlighted that some differences between males and females, stratified by pharmacological groups, are expected. In all age groups, women had a higher prescription prevalence for most pharmacological groups. More particularly, approximately 5% of women received at least one prescription for calcium and drugs for bone structure and this value was higher than that for men (0.4%). It comes as no surprise since the use of antiosteoporosis drugs is a mainly a female issue worldwide [19, 20]. Apart from the greater risk older women run of developing osteoporosis owing to the more extensive loss of bone mineral density [21, 22], the crucial impact of gender may also reflect the greater likelihood of women to obtain a relative drug or supplement with or without prescription.

Another relevant result of our study regarded thyroid preparations. Italian women used these drugs more than men, and the reason is probably to be found in the epidemiological development of thyroid disease [23, 24]. Hoffman F et al. [24] showed that, in Germany, thyroid diseases affect women almost 5 times more frequently than men.

In 2012, Italian women took more antidepressants then men (9.7% women vs 4.5% men). Major depression affects both sexes, but more women than men are likely to be diagnosed with depression in any given year [3, 25]. The prevalence of depression is estimated to be 11.2% in the Italian population, with women using more drugs than men (women 7.8% men vs 3.6%). Women are twice as likely to use antidepressant drugs compared to men.

In addition, such as other international studies, Italian women were undertreated as regards cardiovascular drugs (including antilipidemicemic agents, beta- blockers and related medication and angiotensin – converting enzyme inhibitors). ACE inhibitors primarily used for the treatment of heart failure and hypertension, were more commonly used in Italian men. In this context, in 1991 Bernardine Healy called attention to the discriminatory behavior of cardiologists towards women with under-diagnosed and under-treated ischaemic heart disease (IHD) in a publication in the New England Journal of Medicine entitled “Yentl syndrome” [26]. This disparity between men and women regarding healthcare management appeared to depend largely upon multiple factors related to the patient, to consequences of the disease and to the physician’s assessment of patient risk. Over time, the term ‘Yentl Syndrome’ has come to be used in medicine to define the possibility that diagnostic and therapeutic strategies are not offered in a similar manner to both men and women (or that women are discriminated against). In 2011, Merz CN [27] suggested that the Yentl syndrome is alive and well 10 years later [28, 29]. With regard to this, two new studies demonstrating the medical under-treatment of women, including lower rates of aspirin and ACE inhibitor use in stable women compared to men and lower rates of ACE inhibitor, b-blocker, and statin medication in women with acute coronary syndrome compared to men became available.

It was clear that women in Italy used more drugs then men, and this result is consistent with other drug use statistics. According to data published by the National Agency for Medicines, Italian women have more contact with the healthcare system which may provide them with an opportunity for detecting disease and receiving prescriptions. Women have a higher life expectancy at birth than men (79.4 years men vs. 84.5 years women), suffer from chronic degenerative diseases associated with aging more than men (35.3% men vs 40.0% women), benefit more from the health services and, in the age group between 15 and 64 years in Italy, show a level of drug exposure 8% higher than that of men. Moreover, the fact that most of the preclinical and clinical studies are conducted on male animals and men (gender blindness) and then the results of these studies are then shifted onto women gives rise to most cases of inappropriate therapy. In literature, several papers have highlighted that belonging to one gender rather than to the other represents a risk factor as regards the development of adverse drug reactions (ADR). In fact, female patients have a 1.5 to 1.7 fold greater risk of developing an ADR compared to male patients [30]. In Italy, the data of the National Network of Pharmacovigilance show a greater number (59% in 2011) of spontaneous reports of adverse drug reactions (ADR) in women in all age groups starting from the second year of life. ADRs in women are more numerous and even more serious than in men and lead to a higher number of hospital admissions (about 60% of hospitalizations for ADR regard women) [31]. In contrast with this evidence, a typical antipsychotic prescribed with dementia, psychosis or attention-deficit hyperactivity disorder (ADHD) shows a greater risk of provoking ADRs in male patients than in women [32].

The main strength of this study is the coverage of all dispensed prescription drugs reimbursed by the National Health Service to the entire Italian population. Another strength is the data source of the study which provides a more accurate picture of actual drug use stratified by gender.

The most important limitation of this study is the lack of clinical information on patients in order to assess the reason behind the observed differences. It is important to emphasise that gender differences may only be hypothesised from these data. Moreover, data on out of pocket drugs were excluded. We only had information on the third level ATC, but, in reality, this is not a severe limit since we can identify the pharmacological group by means of the ATC III.

Despite the limitations detailed above, the results of this study made it possible to formulate some thoughts of interest to public health. They represent a starting point for informing health care workers of the importance of gender differences in order for them to provide the best possible health care. In this context, behavourial and preventive interventions would be necessary to reduce the gender disparity. Moreover, the doctor-pharmacist-patient relationship should be characterized by dialogue as an opportunity for education management therapies.

## Conclusions

In this large study, we found substantial differences in drug use between men and women in Italy. The findings may subsequently be used to plan future studies to address differences suggesting inequity in treatment approaches.

Some drug use differences may be explained by variations prevalence and severity of disease, pathophysiology or by other biological differences (due to sex differences). However, it is also evident that other differences need further investigation in order to explain the apparent lack of a rational medical explanation for some findings. A greater awareness of the influence of gender in health and disease is needed to ensure rational drug use in both men and women. Moreover, due to conflicting outcomes between gender differences and ADRs, new surveys in the active pharmacovigilance field should be carried out in order to improve our knowledge of the safety profile of drug use in the treatment of male or female patients.

## Additional file

Additional file 1: Dataset prevalence. Description data: Prevalence data and Risk Ratio. (PDF 11 kb)

## Abbreviations

ACE Inhibitors: Angiotensin-converting-enzyme inhibitor
ADR: Adverse drug reactions
ATC: Anatomical therapeutic chemical
CI: Confidence interval
DDD: Defined daily dose
DDD/1000 inhabitants/day: Defined daily dose per 1000 inhabitants per day
IHD: Ischaemic heart disease
NHS: National Health Service
RR: Risk ratios

## References

[1] Wilson A, McDonald P, Hayes L, Cooney J (1992). Health promotion in the general practice consultation: a minute makes a difference. BMJ.

[2] Soldin OP, Chung SH, Mattison DR (2011). Sex differences in drug disposition. J Biomed Biotechnol.

[3] Regitz-Zagrosek V (2012). Sex and gender differences in health. Science & Society Series on Sex and Science. EMBO Rep.

[4] Gulbins H, Vogel B, Reichenspurner H. Gender effects on health care costs in cardiovascular medicine-a black box? Thorac Cardiovasc Surg. 2013; doi: 10.1055/s-0032-1328931. 10.1055/s-0032-1328931 23225508

[5] Owens GM (2008). Gender differences in health care expenditures, resource utilization, and quality of care. J Manag Care Pharm.

[6] EUGenMed Cardiovascular Clinical Study Group, Regitz-Zagrosek V, Oertelt-Prigione S, Prescott E, Franconi F, Gerdts E, Foryst-Ludwig A, Maas AH, Kautzky-Willer A, Knappe-Wegner D, Kintscher U, Ladwig KH, Schenck-Gustafsson K, Stangl V. Gender in cardiovascular diseases: impact on clinical manifestations, management, and outcomes. Eur Heart J. 2016; doi: 10.1093/eurheartj/ehv598. 10.1093/eurheartj/ehv598 26530104

[7] Campbell CI, Weisner C, Leresche L, Ray GT, Saunders K, Sullivan MD, Banta-Green CJ, Merrill JO, Silverberg MJ, Boudreau D, Satre DD, Von Korff M (2010). Age and gender trends in long-term opioid analgesic use for noncancer pain. Am J Public Health.

[8] Johnell K, Fastbom J (2011). Gender and use of hypnotics or sedatives in old age: a nationwide register-based study. Int J Clin Pharm.

[9] Kautzky-Willer A, Harreiter J. Sex and gender differences in therapy of type 2 diabetes. Diabetes Res Clin Pract. 2017;131:230–41. Epub 2017 Jul 13. 10.1016/j.diabres.2017.07.012 28779681

[10] Klungel OH, de Boer A, Paes AH, Seidell JC, Bakker A (1998). Sex differences in antihypertensive drug use: determinants of the choice of medication for hypertension. J Hypertens.

[11] Stock SA, Stollenwerk B, Redaelli M, Civello D, Lauterbach KW (2008). Sex differences in treatment patterns of six chronic diseases: an analysis from the German statutory health insurance. J Women's Health (Larchmt).

[12] Loikas D, Wettermark B, von Euler M, Bergman U, Schenck-Gustafsson K (2013). Differences in drug utilisation between men and women: a cross sectional analysis of all dispensed drugs in Sweden. BMJ Open.

[13] Ayanian JZ (1999). Using administrative data to assess health care outcomes. Eur Heart J.

[14] Schneeweiss S (2005). Methods in pharmacoepidemiology: an invited series. Pharmacoepidemiol Drug Saf.

[15] Hadji P, Jacob L, Kostev K (2016). Gender- and age-related treatment compliance in patients with osteoporosis in Germany. Patient Prefer Adherence.

[16] Colivicchi F, Gulizia MM, Franzini L, Imperoli G, Castello L, Aiello A, Ripellino C, Cataldo N (2016). Clinical Implications of Switching Lipid Lowering Treatment from Rosuvastatin to Other Agents in Primary Care. AdvTher.

[17] Newman SC (2001). Biostatistical Methods in Epidemiology.

[18] Piovani D, Clavenna A, Cartabia M, Bonati M, Antibiotic Collaborative Group (2012). The regional profile of antibiotic prescriptions in Italian outpatient children. Eur J Clin Pharmacol.

[19] Bor A, Matuz M, Gyimesi N, Biczók Z, Soós G, Doró P (2015). Gender inequalities in the treatment of osteoporosis. Maturitas.

[20] Iolascon G, Gimigliano F, Orlando V, Capaldo A, Di Somma C, Menditto E (2013). Osteoporosis drugs in real-world clinical practice: An analysis of persistence. Aging Clin Exp Res.

[21] Casula M, Catapano AL, Piccinelli R, Menditto E, Manzoli L, De Fendi L, Orlando V, Flacco ME, Gambera M, Filippi A, Tragni E (2014). Assessment and potential determinants of compliance and persistence to antiosteoporosis therapy in Italy. Am J Manag Care.

[22] Cawthon PM (2011). Gender differences in osteoporosis and fractures. Clin Orthop Relat Res.

[23] Morganti S, Ceda GP, Saccani M, Milli B, Ugolotti D, Prampolini R, Maggio M, Valenti G, Ceresini G (2005). Thyroid disease in the elderly: sex-related differences in clinical expression. J Endocrinol Investig.

[24] Hoffmann F, Bachmann CJ, Boeschen D, Glaeske G, Schulze J, Schmiemann G, Windt R (2014). Sex-specific differences in drug utilisation in different phases of life. Bundesgesundheitsblatt Gesundheitsforschung Gesundheitsschutz.

[25] Kautzky-Willer A, Oertelt-Prigione S, Regitz-Zagrosek V (2012). Sex and gender differences in endocrinology. Sex and Gender Aspects in Clinical Medicine.

[26] Healy B (1991). The Yentl syndrome. N Engl J Med.

[27] Merz CN (2011). The Yentl syndrome is alive and well. Eur Heart J.

[28] Johnston N, Schenck-Gustafsson K, Lagerqvist B (2011). Are we using cardiovascular medications and coronary angiography appropriately in men and women with chest pain?. Eur Heart J.

[29] Bugiardini R, Yan AT, Yan RT, Fitchett D, Langer A, Manfrini O, Goodman SG, Canadian Acute Coronary Syndrome Registry I and II Investigators (2011). Factors influencing underutilization of evidence-based therapies in women. Eur Heart J.

[30] Rademaker M (2001). Do women have more adverse drug reactions?. Am J Clin Dermatol.

[31] Franconi F, Campesi I (2014). Sex and gender influences on pharmacological response: an overview. Expert Rev Clin Pharmacol.

[32] Ruggiero S, Rafaniello C, Bravaccio C (2012). Safety of attention-deficit/hyperactivity disorder medications in children: an intensive pharmacosurveillance monitoring study. J Child Adolesc Psychopharmacol.

