# Supplementary material for: Differences in drug use between men and women: an Italian cross sectional study
Source: BMC Womens Health. 2017 Sep 5;17:73. doi: 10.1186/s12905-017-0424-9 (PMC5583764; doi:10.1186/s12905-017-0424-9)
Supplement: Additional file 1: — Dataset prevalence. Description data: Prevalence data and Risk Ratio. (PDF 11 kb) [file 12905_2017_424_MOESM1_ESM.pdf]

ATC code	Pharmacological group	Male	Female	Crude RR (Female/Male)	Age-adjusted RR (Female/Male)	95% Lower bound	95% Upper Bound
**G04C**	Drugs used in benign prostatic hypertrophy	60,5	0,2	0,0	0,0	0,0	0,0
**M04A**	Antigout Preparations	20,4	11,3	0,6	0,4	0,4	0,4
**C01D**	Vasodilators Used In Cardiac Diseases	14,7	14,2	1,0	0,7	0,7	0,7
**C09A**	Ace Inhibitors, Plain	68,6	57,6	0,8	0,7	0,7	0,7
**B01A**	Antithrombotic Agents	111,2	104,6	0,9	0,7	0,7	0,7
**C01B**	Antiarrhythmics, Class I And III	10,3	9,9	1,0	0,7	0,7	0,7
**C02C**	Antiadrenergic Agents, Peripherally Acting	13,1	11,7	0,9	0,7	0,7	0,7
**C08C**	Selective Calcium Channel Blockers With Mainly Vascular Effects	56,3	51,7	0,9	0,7	0,7	0,7
**A10B**	Blood Glucose Lowering Drugs, Excl. Insulins	49,4	44,9	0,9	0,8	0,8	0,8
**R03A**	Adrenergics, Inhalants	32,5	29,7	0,9	0,8	0,8	0,8
**C10A**	Lipid Modifying Agents, Plain	95,5	89,8	0,9	0,8	0,8	0,8
**A10A**	Insulins And Analogues	10,6	10,3	1,0	0,8	0,8	0,8
**R03B**	Other Drugs For Obstructive Airway Diseases, Inhalants	28,0	27,1	1,0	0,8	0,8	0,8
**C09C**	Angiotensin II Antagonists, Plain	45,7	48,1	1,1	0,9	0,9	0,9
**C03C**	High-Ceiling Diuretics	25,8	33,4	1,3	0,9	0,9	0,9
**C07A**	Beta Blocking Agents	70,5	83,3	1,2	1,0	1,0	1,0
**S01E**	Antiglaucoma Preparations And Miotics	16,6	20,8	1,3	1,0	1,0	1,0
**C09B**	Ace Inhibitors, Combinations	35,3	44,0	1,2	1,0	1,0	1,0
**N03A**	Antiepileptics	18,3	21,3	1,2	1,1	1,1	1,1
**A02B**	Drugs For Peptic Ulcer And Gastro-Oesophageal Reflux Disease (Gord)	125,2	161,2	1,3	1,1	1,1	1,1
**C09D**	Angiotensin II Antagonists, Combinations	45,2	61,1	1,4	1,1	1,1	1,1
**R06A**	Antihistamines For Systemic Use	20,3	25,9	1,3	1,3	1,3	1,3
**H02A**	Corticosteroids For Systemic Use, Plain	22,7	32,4	1,4	1,3	1,3	1,3
**J01C**	Beta-Lactam Antibacterials, Penicillins	52,1	69,9	1,3	1,3	1,3	1,3
**M01A**	Antiinflammatory And Antirheumatic Products, Non-Steroids	72,2	122,4	1,7	1,5	1,5	1,5
**B03B**	Vitamin B12 And Folic Acid	3,6	6,9	1,9	1,6	1,6	1,6
**N06A**	Antidepressants	28,8	66,8	2,3	2,0	2,0	2,0
**B03A**	Iron Preparations	3,8	9,9	2,6	2,1	2,1	2,1
**H03A**	Thyroid Preparations	11,7	63,7	5,4	5,0	5,0	5,0
**A12A**	Calcium	2,8	23,9	8,6	7,0	7,0	7,0
**M05B**	Drugs Affecting Bone Structure And Mineralization	2,0	31,2	16,0	12,5	12,5	12,5
